# Editorial: Promoting brain health and emotional regulation through prosocial behaviors, social connectedness, and AI

**DOI:** 10.3389/fnhum.2026.1848176

**Published:** 2026-04-21

**Authors:** Nayara Mota

**Affiliations:** 1 Institute of Psychology and Social Sciences, Federal University of Bahia (UFBA), Salvador, Brazil; 2 School of Psychology, Catholic University of Salvador (UCSal), Salvador, Brazil

**Keywords:** artificial intelligence, brain health, emotional regulation, prosocial behavior, social connectedness

Mediation has long been recognized as a central mechanism in human development, as proposed by Vygotsky (2012). However, contemporary advances in social neuroscience (Legaz et al., 2026) and Artificial Intelligence (AI) (Han et al., 2026) suggest that mediation should no longer be understood merely as a contextual support for development, but as a dynamic process that actively shapes brain functioning. In this sense, social interaction and AI-mediated processes emerge not only as protective factors, but also as foundational elements of neurocognitive organization, whether naturally occurring or artificially implemented. This convergence has increasingly attracted scholarly attention, advancing social neuroscience and fostering the emergence of neural AI. As a contribution to this growing field, this Research Topic aims to explore the links between social connection, prosocial behavior, and neuropsychological outcomes, whether or not AI-based approaches are involved.

Addressing this intersection requires a more precise understanding of how prosocial behavior operates. Rather than treating prosociality as a unitary construct, Kim and Sul advance the field by distinguishing between healthy and unhealthy impacts of prosocial behavior. This distinction is not trivial: it suggests that prosocial behavior may rely on qualitatively different neurocognitive mechanisms, with distinct implications for brain health. The neural systems underlying prosocial behavior involve the activation of primary and more responsive brain regions, including the ventral tegmental area and the insula, as well as later-developing and self-regulating regions, including the ventral striatum, cingulate cortex, and the medial prefrontal cortex. Moreover, Kim and Sun propose four dynamics linking prosocial behavior to physical health. Such a perspective calls for a re-examination of the assumption that all prosocial engagement is inherently beneficial and opens new directions for investigating differential neural activation underlying the same type of behavior.

A similar conceptual refinement is needed in the study of social connectedness. Different levels of social engagement have been differentiated by Hajek et al., who associate hugging with loneliness, social isolation and social withdrawal. In a German national survey of 3,270 adults and seniors, the non-verbal prosocial behavior of hugging was associated with all three measurements indicative of social connectedness. The transition from prosocial behavior to social connectedness remains to be elucidated. Early neuropsychological evidence suggests that prosocial dispositions can optimize adaptive engagement with the environment, potentially anticipating more complex forms of social connectedness. In a contextualized statistical analysis incorporating psychosocial and sociodemographic factors, kindness was associated with all examined components of executive functioning (Mota et al., 2017). Current literature on social connectedness, although still incipient in social neuroscience, suggests that reciprocity and non-verbal communication trigger neurocognitive development, especially for those under conditions of vulnerability (Mota, 2025). However, the dynamics linking social connectedness with brain health and emotional regulation remain insufficiently explored. Addressing this gap is essential for advancing a more unified framework regarding dynamically co-constitutive processes underlying brain health.

These contributions have direct implications for promoting emotional regulation. Social interaction facilitates the transition from immediate emotional reactivity to more regulated responses. Interestingly, both prosocial behavior and emotional expression share activation in the aforementioned brain regions. Primary brain regions, including the insula, are implicated in emotional reactivity; while later-developing regions, including the medial prefrontal cortex, support regulated emotional responses (Luo, 2018). This transition relies on the ability to filter implicit contingencies, integrate multiple sources of information, and generate adaptive interpretations and reactions in real time, thereby engaging a broad neural network. Thus, social connectedness can be conceptualized as a dynamic adaptive process underlying emotional regulation, constituting an emergent property of relational and neural dynamics rather than an isolated individual capacity.

Within this framework, prosocial behavior can also be fostered in routine professional practice, optimizing emotional regulation and health-related outcomes. In an experimental approach with 50 Chinese oncology patients, Pan et al. demonstrated that enhanced comprehensive nursing positively influenced pain, mood, and quality of life. Extending the study of prosocial behavior from a conceptual framework to a clinical setting strengthens the evidence of its beneficial impact on wellbeing.

Taken together, these conceptual and experimental contributions on the interplay between prosocial behavior, social connectedness, and emotional regulation highlight the need for further interdisciplinary investigation into how social dynamics interact with brain activation, shaping emotional and neurocognitive resources. They also point to the need for a fundamental reconsideration of brain health: rather than being defined solely in terms of internal neural states, brain health can be understood as emerging from the continuous interaction between preverbal interactions, relational processes, emotional expression, and cognitive representations. Thus, social interaction and connectedness sustain a flow of sensory and non-verbal information that supports coordinated and efficient brain activation.

Complementing natural social mediation, AI provides an additional layer by externalizing and structuring emotional and relational signals. Through mechanisms like facial recognition and pattern detection, AI translates non-verbal information into verbal and conceptual formats, supporting cognitive appraisal and other strategies for emotional regulation. Importantly, AI is not a substitute for human interaction but a tool that makes implicit processes more accessible and easier to regulate. A promising contribution of AI resources to support human social cognition has been presented by Street et al. Comparing the performance of Large Language Models (LLMs) with 29,259 UK-based participants, the authors identified that GPT-4 and Flan-PaLM reached adult-level or near-adult-level ability to theorize about others' minds, across different levels of complexity. However, the potential benefits of AI as a tool for neurocognitive stimulation and timely support must be weighed carefully against the risks associated with using Natural Language Generation (NLG) for persuasion and manipulation.

In this context, advancing the field requires more than accumulating empirical findings. It calls for a conceptual framework capable of articulating how sensory signals processed in social or AI-mediated contexts are organized into both higher-order cognitive representations and adaptive patterns of emotional and relational experience. Emotional and brain health can be understood as forms of experiential wellbeing shaped by social interaction, encompassing prosocial behavior, social connectedness, and the ethical use of AI-based tools, which function as socially derived mediational resources for human development.
